# Life as an early career researcher: interview with Heloisa Helena Milioli

**DOI:** 10.4155/fsoa-2016-0033

**Published:** 2016-06-06

**Authors:** Heloisa Helena Milioli

**Affiliations:** 1 Priority Research Centre for Bioinformatics, Biomarker Discovery & Information-Based Medicine, Hunter Medical Research Institute, New Lambton Heights, NSW, Australia; 2 School of Environmental & Life Science, The University of Newcastle, Callaghan, NSW, Australia

**Keywords:** bioinformatics, breast cancer, early career, intrinsic subtypes

## Abstract

**Heloisa Helena Milioli speaks to Francesca Lake, Managing Editor:** Heloisa received a BSc degree in Biological Sciences (2008) from the Universidade Federal de Santa Catarina (Brazil) and obtained a MSc degree in Genetics (2011) from Universidade Federal do Paraná (Brazil). In 2011 and 2012, she worked as a lecturer and tutor in the Department of Cell Biology, Embryology and Genetics (Universidade Federal de Santa Catarina). She moved to Australia in 2012 to obtain her PhD in Biological Sciences, with emphasis on Bioinformatics, from The University of Newcastle. Her doctoral work brings together new considerations in the breast cancer field by combining novel bioinformatics approaches with the study of intrinsic subtypes. She has been applying advanced methods and sophisticated algorithms in unconventional computer architecture for the molecular classification of breast cancer based on the genomic (single nucleotide polymorphisms, circulating nucleic acids and copy number variations) and transcriptomic (gene expression and miRNA) signatures. Fundamental research will allow her to identify biomarkers of use in translational medicine for the diagnosis, prognosis and disease management focused on group-based tailored therapies.

## Q Can you tell us about your career path to date?

I began my academic career with a bachelor's degree in Biological Sciences at the Universidade Federal de Santa Catarina (UFSC), Brazil. While studying, I worked as an intern at the ‘Department of Molecular Biology, Embryology and Genetics’ performing cytogenetic (classical and molecular) analysis of breast tumors and hematological disorders. I completed my bachelor's in August 2008, presenting original research in the form of a monograph as part of the requirements for the degree. At that time, I could not imagine myself far from academia. I have always had a keen interest in learning and a strong desire to develop new knowledge. I then decided to pursue a master's degree in Genetics the following year, at Universidade Federal do Paraná (UFPR), Brazil. During my postgraduate studies, I directed my research toward hypothesis-driven proteomic analysis of breast cancer; obtaining my degree in 2011. In the same year, I had the opportunity to work as a ‘Substitute Professor’ at UFSC, in Brazil. After being selected through a highly competitive scheme, I lectured on Genetics, Molecular Biology and Evolution for several courses. It was a markedly rewarding experience which encouraged me to continue in academia. I left my country, taking up a highly competitive and well regarded doctoral scholarship in Australia (in an English-speaking environment).

I am currently enrolled full-time in the Faculty of Science and Information Technology PhD program in Biological Sciences at The University of Newcastle, Australia. In my PhD project, I have applied integrated bioinformatics methodologies to analyze large-scale genomic and transcriptomic data. Accordingly, I have explored the prominent dataset processed by Molecular Taxonomy of Breast Cancer International Consortium (METABRIC) [1], containing over 2000 samples, with the aim of refining and expanding the understanding of breast cancer intrinsic subtypes. This exposure to the field of bioinformatics has greatly extended my expertise, both personally as a scientist and by expanding my skillset and enhancing my versatility and ability to embrace future opportunities. I have a particular interest in the translation of fundamental/basic knowledge – from the identification of biomarkers for diagnostics, prognostics and therapeutics – into clinical applications focused on individualized/personalized medicine.

## Q Can you tell us more about what your current position entails, & what you are working on?

In my current position, I work on the analysis of the METABRIC breast cancer dataset. I use mainly the R programming language and Bioconductor packages for the analysis and comprehension of high-throughput microarray data and clinical sources. Although I began with a basic knowledge of statistics (Statistics for Biological Sciences), my natural curiosity and love of learning has allowed me to develop skills to the level required for the complex data analytic tasks inherent in this work. All knowledge acquired has undoubtedly facilitated a more efficient and effective incorporation of complementary approaches – bioinformatics resources, medical libraries and clinical research – to address health issues in the era of ‘Big Data’. One of the most important achievements of my PhD was the identification of inconsistent intrinsic subtype labels assigned in the original METABRIC discovery and validation datasets [2,3]. Our group identified the problem and devised an innovative approach to address the issue, providing a set of reliable biomarkers and an ensemble learning approach able to differentiate the breast cancer subtypes. A more reliable classification is also available for researchers in the field who wish to work with consistent and homogeneous groups and avoid discrepancies in research moving toward clinical applications.

## Q Where do you see yourself in 10 years?

I am a dreamer. I see myself working in academia with a well-established career; with a secure job and a decent salary. In my ideal scenario, I see myself surrounded by researchers from different areas (biology, chemistry, physics, mathematics, statistics, computer science, medicine and psychology), collaborating for a cancer ‘moonshot’ endeavor [4]. There is no pressure from government, academia or the private sector to do anything but produce high-quality translational science. The multidisciplinary, dynamic environment is less destructively competitive and more productive. Researchers share data, publish less quantity and more quality papers and are evaluated based on patient outcomes.

Back to reality, back to life! It is not easy to find stability in academia. In the current landscape, researchers are not incentivized to share data or stimulated to think ‘outside the box’, which may be the answer to some cancers. Grants are given to senior academics based on what they have done instead of what researchers (early-mid career) could be doing. Even though everyone works as part of a team – and groups tend to innovate faster, see mistakes more quickly and find better solutions to problems [5] – everyone is also ‘out for themselves’. This means that researchers are responsible for their own success. There is yet a worse and cruel scenario where researchers are exploited with low paid and insecure work, or working for free (unpaid internships), in return for valuable skills and experience or a name on a paper.

The truth is: I can barely see myself next year. However, I am 150% sure I want to stay in academia today [6], not sure about tomorrow!

## Q What do you find most rewarding about your work?

What I find most rewarding about academia is the constant learning, exploration of new ideas and sharing with intellectual peers. Independence!

It has also been rewarding and interesting to work on different approaches (cytogenetic techniques, genomic and transcriptomic data analysis, proteomic access and bioinformatics applications) in breast cancer research. In parallel, I have learned other intangible valuable skills like how to process and present information, integrate knowledge, think strategically and collaborate with other researchers. Working in a multidisciplinary team environment also allowed me to conciliate research programs to life endeavors. I am lucky to be part of an ethnically diverse group (Australian, Bangladeshi, Brazilian, Chilean, Indian, Iranian, Jordanian, Dutch and Swiss) where I learn every day. My career path has been extremely pleasing, and I feel fortunate.

## Q What would you say are the biggest challenges facing early-career researchers, from your point of view?

The PhD is just the beginning. The first ‘big’ challenge is to find the ‘perfect’ postdoctoral position. Every year, most scientists have to deal with the uncertainty of the following year; of suddenly having no salary, no job and no career. If you are lucky enough to have a supervisor that supports you, emotionally and financially, this is a good start. If not, as in most cases, you will have to apply for distinct positions and funding – grants and fellowships – and be ready to change topic and institution. Like almost all transitions, it is never easy! Those who better accept the changes I believe will persevere and continue doing research, and ‘survive’ in academia. I expect I will be one of them! It requires not only good ideas but an ability to sell the ideas, which is something that requires practice and constant improvement. The ultimate challenge is to find a permanent position, build your own laboratory and research group and keep innovating.

In the near future, I will be looking for positions (lecturer, research assistant and post-doctoral researcher) and applying for funding everywhere. It means that perhaps I will move university, city, state or country. This ‘change’, nevertheless, is not only mine, it will involve my family. I know that it is important to learn from and explore the diverse environment and culture at every institution, but other factors also need to be considered: the costs of moving, the time for adapting and the chances of stability. It can be hard, for both males and females, when you have a family, with other values to balance: the quality of life, the health system, the education scheme, among others. Even harder if you are a mother or plan to have children. Despite recent gains in closing the scientific gender gap, female researchers around the world continue to face extra challenges (lower salaries and less institutional support) and abandon academia at twice the rate as men [7]. As a female scientist, I expect to see a new generation of scientists transforming this scenario and building a healthier environment of ‘survivors’.

## Q How would you suggest these are tackled?

Never wait for someone to give you a chance to learn or gain experience, create your own opportunities and have your own experiences. More importantly, find a way to connect them. You will increase your chances of having great ideas of what you would like to do and how you would like to conduct your research in the future. Learn as much as you can from different methods, instruments and technologies. These evolve so quickly you cannot rely on the specific, concrete skills you learnt at university. Learn from the challenges associated with the PhD period; enjoy the years spent gaining post-doctoral experience before finding a permanent academic role. Have in mind that as a mature, educated and committed worker you are of tremendous value not only to academia, but to other organizations outside the field as well. Keep it as a ‘plan B’. Having a ‘plan B or C’ will help you to build self-confidence in the scientific environment and beyond. Mainly, be prepared to change!

Participate in scientific events. It is your chance to meet and interact with renowned researchers from all over the world. Present your work to the right person, at the right time and in the right event. Communicate, exchange opinions, build solid connections and seed future collaborations with other students and professionals in the field. Engage with researchers that you feel comfortable with and find mentors to support you throughout the academic career. Promote teamwork and learn how to work in collaboration. The benefits of teamwork are unlimited: distinct ideas (brainstorming), aims and deadlines, smart strategy, shared expertise, high quality, focus, strength, support and satisfaction, etc. Perhaps you will not be able to tackle all the upcoming challenges, but most will yield.

## Q What advice would you give someone who is just starting out in an undergraduate degree, & wants to go into academia?

A career in academia can be very rewarding, with unique opportunities and challenges. My main advice is: experience everything to make sure you want to take this path. Seek advice on ‘alternative’ careers, inside and outside academia. Look for your ‘perfect’ positions (at least the ones that match your perspectives). Think about your goals and expectations: What is more important for you? Advisor relationship? Teamwork? Publications? University reputation? Research topic? A lot of these questions will have to be answered truthfully and in-depth during your PhD, and are things you must find out yourself (the sooner, the better). What you can do upfront is to talk with the research group and make sure you are selecting the right advisor, team, university or topic. Be strategic and aware of what is coming ahead.

Be passionate about your work and enjoy learning. I am not saying that you need to be happy all the time and not have crises (I just had one answering the previous questions). People react in a different way in the research environment, especially with the ups and downs of a PhD. At least, you are surrounded by individuals who are in the same situation and completely understand your frustrations. Work collaboratively, but also plan your research and design your experiments. At the end, step up and make your own decision. And do not dream too much!
